# Potentiation Increases Peak Twitch Torque by Enhancing Rates of Torque Development and Relaxation

**DOI:** 10.2478/hukin-2013-0048

**Published:** 2013-10-08

**Authors:** Christian Froyd, Fernando Gabe Beltrami, Jørgen Jensen, Timothy David Noakes

**Affiliations:** 1 UCT/MRC Research Unit for Exercise Science and Sports Medicine, Department of Human Biology, University of Cape Town, South Africa.; 2 Faculty of Teacher Education and Sport, Sogn og Fjordane University College, Norway.; 3 Department of Physical Performance, Norwegian School of Sport Sciences, Norway.

**Keywords:** Maximal isometric voluntary contraction, quadriceps muscles, electrical stimulation, decay, fatigue

## Abstract

The aim of this study was to measure the extent to which potentiation changes in response to an isometric maximal voluntary contraction. Eleven physically active subjects participated in two separate studies. Single stimulus of electrical stimulation of the femoral nerve was used to measure torque at rest in unpotentiated quadriceps muscles (study 1 and 2), and potentiated quadriceps muscles torque in a 10 min period after a 5 s isometric maximal voluntary contraction of the quadriceps muscles (study 1). Additionally, potentiated quadriceps muscles torque was measured every min after a further 10 maximal voluntary contractions repeated every min (study 2). Electrical stimulation repeated several times without previous maximal voluntary contraction showed similar peak twitch torque. Peak twitch torque 4 s after a 5 s maximal voluntary contraction increased by 45±13% (study 1) and by 56±10% (study 2), the rate of torque development by 53±13% and 82±29%, and the rate of relaxation by 50±17% and 59±22%, respectively, but potentiation was lost already two min after a 5 s maximal voluntary contraction. There was a tendency for peak twitch torque to increase for the first five repeated maximal voluntary contractions, suggesting increased potentiation with additional maximal voluntary contractions. Correlations for peak twitch torque vs the rate of torque development and for the rate of relaxation were r^2^= 0.94 and r^2^=0.97. The correlation between peak twitch torque, the rate of torque development and the rate of relaxation suggests that potentiation is due to instantaneous changes in skeletal muscle contractility and relaxation.

## Introduction

Skeletal muscle torque production in response to a single stimulus (SS) of electrical stimulation – the peak twitch torque – is increased after a brief isometric maximal voluntary contraction (MVC) (Vandervoort et al., 1983; Green and Jones, 1989; Hamada et al., 2000; Miyamoto et al., 2010). This phenomenon is known as potentiation or post activation potentiation (Krarup, 1981; Rassier and Macintosh, 2000). Potentiation is probably an important physiological phenomenon that could impact on performance during high intensity exercise (Sale, 2002). It is important to understand the nature of potentiation because peak twitch torque in response to electrical or magnetic stimulation is commonly used to quantify changes in muscle function during and after exercise, which in itself can cause potentiation (Kufel et al., 2002; Place et al., 2010).

An important component is to understand how peak twitch torque is affected by potentiation and the duration of MVC to obtain maximal potentiation. Previous studies have shown that the ideal MVC duration to maximize potentiation appears to be specific to each muscle group (Vandervoort et al., 1983). Most previous studies have used 7–10 s MVCs to characterize the decay of potentiation of the quadriceps muscles (Requena et al., 2008; Paasuke et al., 2007; Folland et al., 2008; Green and Jones, 1989; Hamada et al., 2000). However, 5 s MVCs are more commonly used to potentiate the quadriceps muscles during or after exercise (Romer et al., 2007). Recently, Miyamoto et al. (2012) reported that an MVC of 5 s duration was sufficient to obtain full potentiation of the quadriceps muscles. Furthermore, 5 s MVC did not develop fatigue, which is advantageous compared to when 10 s MVC was to potentiate the peak twitch torque (Miyamoto et al., 2012). Potentiation decays rapidly after an MVC, but after MVCs of 7–10 s of the quadriceps muscles, potentiation is still significant after 5 min (Hamada et al., 2000; Paasuke et al., 2007; Requena et al., 2008; Folland et al., 2008). The decay in peak twitch torque at rest after isometric MVCs of 5 s of the quadriceps muscles has not been studied, although the shorter MVC may be superior to 7–10 s MVCs to potentiate peak twitch torque in exercise studies.

Repeated 5 s MVCs of the quadriceps muscles are used to measure baseline neuromuscular function prior to exercise, but it is still unknown whether 5 s MVCs repeated every min will increase or decrease peak twitch torque further after the first MVC. Therefore, repeated 5 s MVCs of the quadriceps muscles should be performed to understand the effect of these measurements on development of potentiation, decay of potentiation and fatigue.

Therefore, the first aim of the study was to measure the extent to which changes in peak twitch torque and other mechanical properties in skeletal muscle occur in response to 5 s MVC. We measured changes in the rate of torque development and relaxation to evaluate potential changes in skeletal muscle contractility and relaxation that might explain the potentiation phenomenon. The second aim was to investigate if repetitive MVCs performed once every min would influence peak twitch torque and decay of potentiation.

## Material and Methods

The study was approved by the Research and Ethics Committee of the Faculty of Health Science of the University of Cape Town and all experiments were performed according to the latest (2008) revision of the Declaration of Helsinki. Subjects gave their written informed consent to participate in the study after completing a health screening questionnaire. Subjects were given a full explanation of the details and rationale of the study and were informed that they were free to withdraw from the study at any time. The possibility that electrical stimulation might cause discomfort was fully explained as it was the nature of the risks involved.

### Participants

In total 11 subjects participated in the 2 studies presented in this article. Five physically active (> 5 times a week) subjects (2 women and 3 men), whose average (± SD) age, body mass and height were 22.9 ± 4.0 years, 70.8 ± 7.4 kg and 175.8 ± 9.4 cm, respectively, volunteered to participate in study 1. Six physically active (> 5 times a week) subjects (1 women and 5 men), whose average (± SD) age, body mass and height were 21.8 ± 1.0 years, 77.2 ± 9.2 kg and 178.5 ± 10.4 cm, respectively, volunteered to participate in study 2. Subjects refrained from vigorous exercise the day before testing and did not exercise on the day of testing. Nor did they ingest alcohol, coffee or other stimulants on the day of testing.

### Protocol

The subjects visited the laboratory on one occasion for each study. Subjects were secured to a Biodex System 3 isokinetic dynamometer (Biodex Medical System, Shirley, NY, USA) by chest and hip strapping to avoid excessive lateral and frontal movements. The seating was adjusted for each subject with the right knee femoral epicondyle aligned with the axis of the rotation arm of the dynamometer. The right lower leg was attached to the lever arm just above the lateral malleolus. The knee and hip angles were positioned at 90 and 110 degrees, respectively, during all experiments. The left leg was not active and was secured to the dynamometer by strapping around the upper leg. During both the MVC and electrical stimulation, subjects maintained their hands in the same position by holding the chest strapping of the dynamometer.

### MVC

Subjects performed 5 s isometric MVC of the right knee extensors. They were instructed to reach maximum torque in 1 s and then to maintain this level for 4 s. They received strong verbal encouragement to maintain a maximal effort during all contractions while they received constant visual feedback of their torque production. Torque was measured in the isokinetic dynamometer for both the MVC and electrical stimulation assessment.

### Electrical stimulation

After identification of the femoral nerve by palpation, SS electrical stimulation was applied percutaneously with a ball probe cathode pressed manually onto the femoral nerve. The anode, a 130x80 mm self-adhesive electrode (Cefar-Compex Scandinavia AB, Sweden), was applied to the gluteal fold.

A constant current stimulator (DS7AH, Digitimer, Hertfordshire, United Kingdom) delivered a square-wave stimulus of 200 μs duration at maximum 400 V. The optimal stimulation intensity was determined by increasing the current gradually from 10 mA until a plateau in torque (50–115 mA) was reached. The current was then increased by further 30% (70–150 mA) to ensure supramaximal stimulation. The stimulation intensity was kept constant for the same subject during the whole experiment. The subjects were instructed to relax fully when electrical stimulation was applied. In both studies the initial electrical stimulation was first performed only after the subject had rested in position in the dynamometer for 10 min without moving the leg that was to be tested. Electrical stimuli and torque were recorded at 2000 Hz and synchronized by using the AcqKnowledge data analysis software (MP150; Biopac System, Santa Barbara, CA).

### Study 1

**Figure 1:**
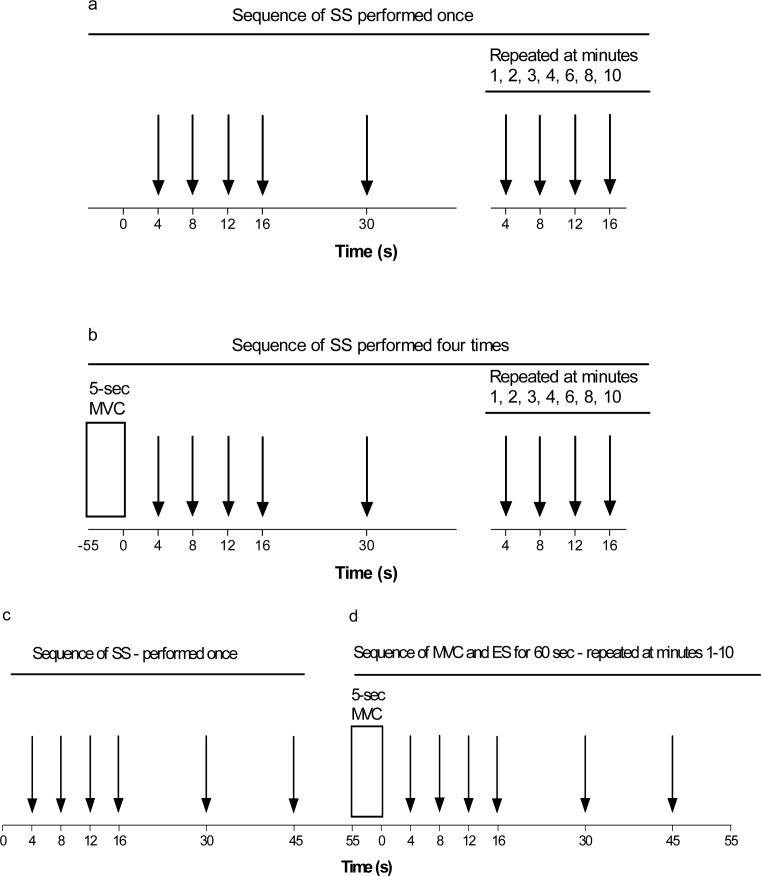
Study 1 consisted of electrical stimulation delivered during 10 min at rest (a) whereas the next 10-min sequence, performed 4 times, followed the same protocol but after an initial 5 s MVC (b). The total duration of study 1 was approximately 54 min. In study 2, electrical stimulation was initially delivered 6 times in 1 min at rest (c), and then at 4, 8, 12, 16, 30 and 45 s after an MVC (d). At every min repeated MVCs with following electrical stimulation (d) was performed, for a total of 10 repetitions. Each arrow indicates a single stimulus. MVC, maximal voluntary contraction. SS, single stimulus.

SS was applied at 4, 8, 12, 16 and 30 s after the start of the experiment. After 1, 2, 3, 4, 6, 8 and 10 min SS was applied at 4, 8, 12 and 16 s (Figure 1a). Thus, the time course of torque development in response to electrical stimulation was measured at 33 time points in the 11^th^ min of this section of the experiment performed on unpotentiated, rested muscle.

To study the effect of a single MVC on potentiation and its decay, subjects performed an identical sequence (presented in Figure 1a) with a preceding 5 s MVC. This sequence of MVC and the following SS (Figure 1b) were performed 4 times and each sequence began 11 min after the start of the previous sequence. To carefully characterize decay of peak twitch torque after MVC, high numbers of stimuli were used (Figure 1b). Identical stimulation protocol was used in control situation (Figure 1a).

### Study 2

Figure 1c shows the time course of the sequence at which SS was delivered to the leg muscles in this section of the experiment. SS was first applied to the rested leg at 4, 8, 12, 16, 30 and 45 s. Thereafter between 55–60 s subjects performed 5 s MVC followed by SS again at 4, 8, 12, 16, 30 and 45 s (Figure 1d). This sequence was then repeated each min for a further 9 min until a total of 10 MVCs with subsequent electrical stimulation was performed. Six episodes of electrical stimulation in 45 s were performed to measure the decay in PT several times.

### Experimental variables and data analysis

The mechanical responses to electrical stimulation are reported as torque. The SS torque response was analyzed to determine peak twitch torque (PT), contraction time (CT), which is the time from start of the contraction to PT, electromechanical delay (EMD), which is the time from electrical stimulation to start of the contraction, the rate of torque development (RTD), which is PT/CT, half relaxation time (½RT), which is the time from PT to 50% decline in PT, and the rate of relaxation (RR) which is PT/½RT.

### Statistical analyses

The data were analyzed with Statistica 10.0 (Stat Soft. Inc., Tulsa, OK, USA). Descriptive statistics are presented as means ± SD. For MVC and peak twitch torque two way repeated measures ANOVA (torque × time) were used to detect differences over time. For CT and ½RT two way repeated measures ANOVA (duration × time) were used to detect differences that could occur over time. For RTD and RR two way repeated measures ANOVA (torque development × time) were used to detect differences over time. The Tukey *post hoc* test was used to determine the specific differences between post-MVC values, or between the values after the 10 MVCs. Linear correlations between percent change in PT and RTD, and PT and RR were performed. The differences between unpotentiated and potentiated measurements were analyzed with the t-test. The statistical significance was defined at p<0.05.

## Results

Average peak torque (± SD) for the 4 MVCs in study 1 and the 10 MVCs in study 2 was 247.5 ± 91.6 Nm and 270.3 ± 75.0 Nm, respectively. There was no significant (p>0.05) difference in peak torque of the MVCs 1–4 in study 1 or of the MVCs 1–10 in study 2.

### Study 1 Unpotentiated muscle: Torque response to repeated SS over 10 min

Repeated SS during the 10 min period (Figure 1a) did not cause any changes (p>0.05) in the mechanical responses, and mean values for PT, EMD, CT, ½RT, RTD and RR were respectively 43.2 ± 7.3 Nm, 31.7 ± 7.1 ms, 89.2 ± 13.3 ms, 76.0 ± 9.7 ms, 0.49 ± 0.11Nm/ms and 0.58 ± 0.17 Nm/ms (Table 1).

### Potentiated muscle: Torque response to repeated SS after one MVC

**Figure 2:**
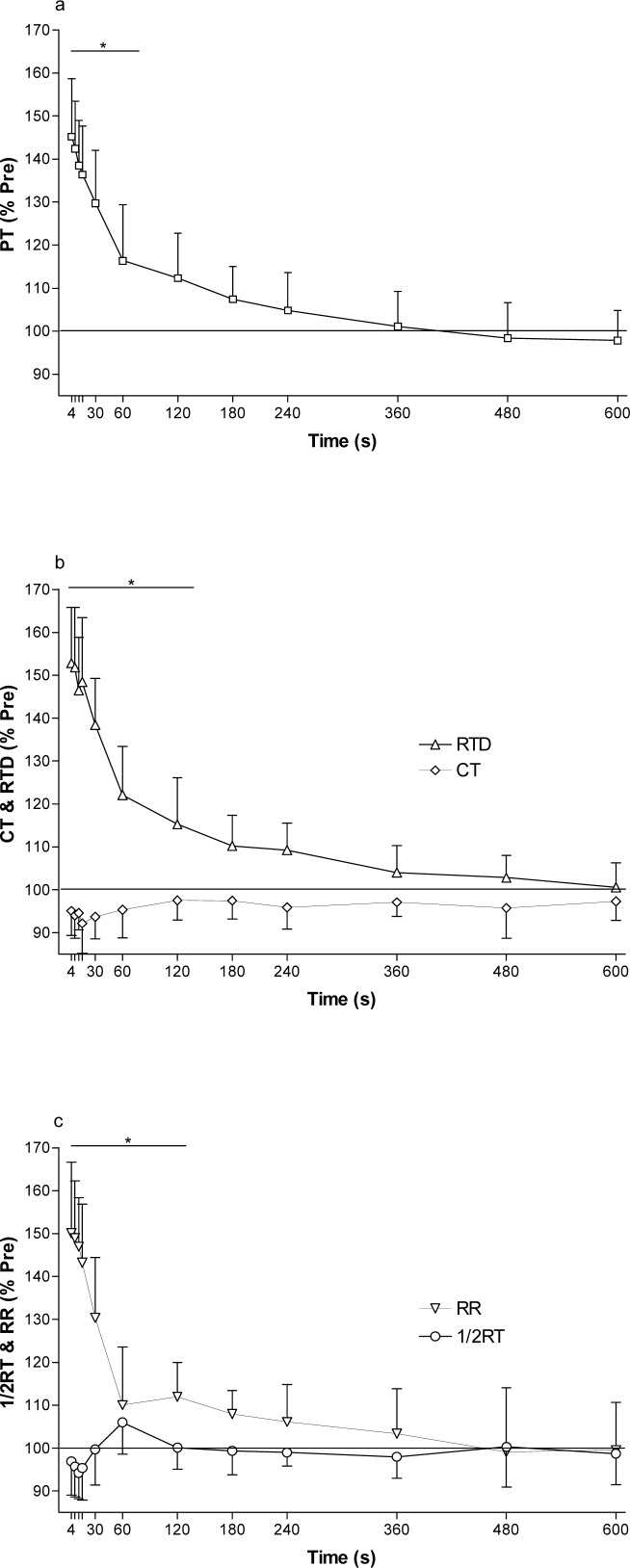
Time decay of PT (a), RTD & CT (b), and RR & ½RT (c) after a 5 s MVC in response to electrical stimulation reported as % change from unpotentiated values for study 1. * p< 0.05 for unpotentiated values. PT, peak twitch torque. CT, contraction time. RTD, rate of torque development. RR, rate of torque relaxation. ½ RT, half relaxation time.

After a single MVC (Figure 1b), there were no significant differences (p>0.05) in SS mechanical responses between the 4 sequences of 10 min of stimulation. Therefore, results for the SS mechanical responses for these 4 sequences were averaged for each subject and were analyzed together (Figures 2a, 2b and 2c). Four seconds after the initial MVC, PT was 62.6 ± 10.8 Nm, a 45 ± 13% increase compared to the pre-MVC value (Figure 2a). There was a sharp decline in PT in the following 60 s so that PT after 2 min was not significantly different (p>0.05) from the pre-MVC PT (Figure 2a). However, PT returned to baseline pre-MVC value only after 6 min.

RTD and RR increased significantly (p<0.05) by 53 ± 13% and 50 ± 17%, respectively, immediately after the MVC whilst CT and ½RT were unchanged for the duration of the experiment (Figures 2b and 2c). RTD and RR returned to the pre-MVC values within 3 min after the initial MVC. The decay in PT was associated with a progressive fall in the RTD and in the RR (Figures 2b and 2c). Correlation between PT vs RTD, PT vs RR and PT vs CT was r^2^ = 0.99 (p<0.001), 0.98 (p<0.001) and 0.56 (p<0.01), respectively, during the 10 min period after the MVC. EMD did not change at any time during this section of the experiment (data not shown).

### Study 2 Unpotentiated muscle: Torque response to repeated SS over 1 min

SS torque response to the first 6 episodes of electrical stimulation (Figure 1c) delivered to the unpotentiated muscle in the min prior to the first MVC did not differ from each other (p>0.05) and the mean values did not differ from those of study 1. Mean values for PT, EMD, CT, ½RT, RTD and RR were respectively 43.5 ± 12.9 Nm, 34.2 ± 3.1 ms, 85.9 ± 9.5 ms, 80.3 ± 10.5 ms, 0.52 ± 0.18 Nm/ms and 0.56 ± 0.21 Nm/ms (Table 2).

### Potentiated muscle: Torque response to repeated SS after 10 MVCs

**Figure 3:**
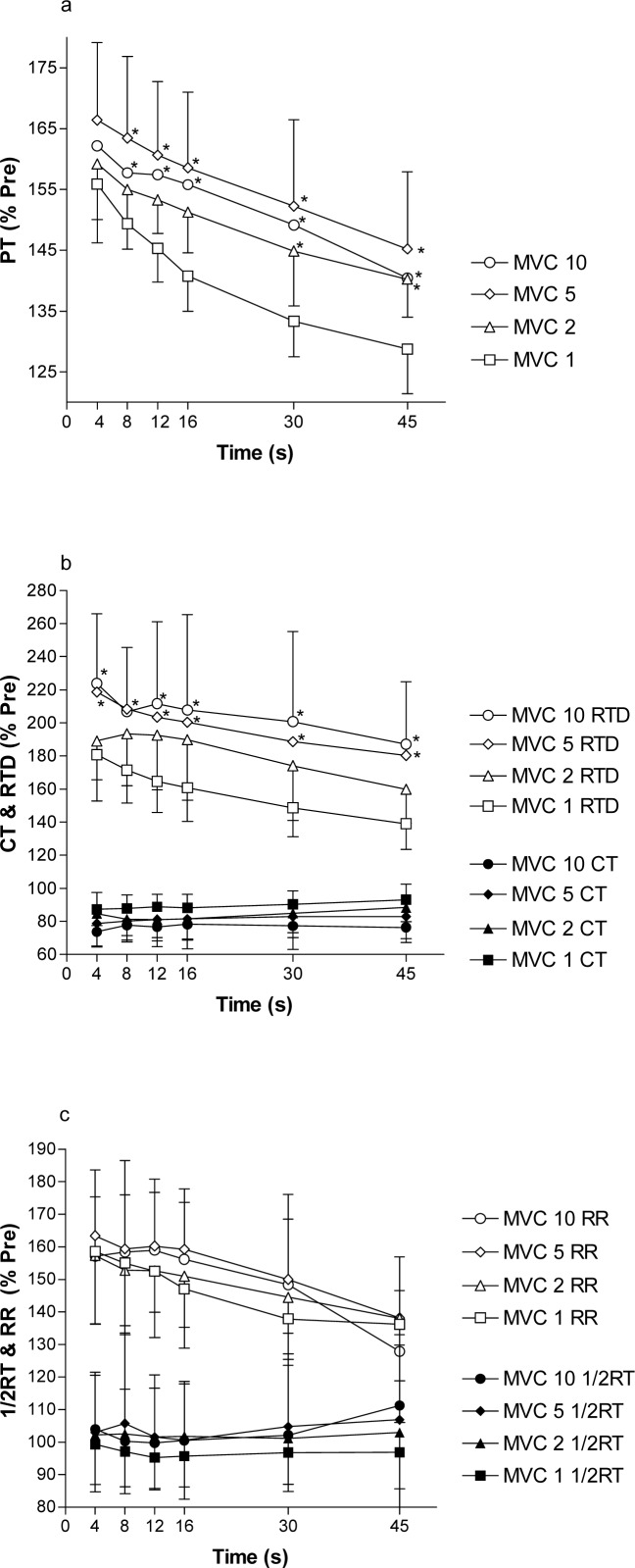
Time decay of PT (a), RTD & CT (b) and RR & ½RT (c) after a 5 s MVC in response to electrical stimulation reported as % change from unpotentiated values for study 2. * p< 0.05 from MVC 1. Other values were not different between MVCs. All PT-, RTD- and RR-values are significant different (P<0.05) from unpotentiated values. All CT-values except the 5^th^ and 6^th^ measurement (30 and 45 s) for MVC 1 are significantly different (p<0.05) from unpotentiated values. MVC, maximal voluntary contraction. PT, peak twitch torque. CT, contraction time. RTD, rate of torque development. RR, rate of torque relaxation. ½RT, half relaxation time.

PT immediately (4 s) after the first MVC (MVC 1) was increased by 56 ± 10% (Figure 3a) to 67.0 ± 17.7 Nm. PT immediately after MVCs 2–10 was not different (p>0.05) from PT immediately after MVC 1 (Figure 3a).

PT then decayed from 4–45 s after each MVC so that at 16 s after MVC 1, PT fell significantly (p<0.001) from the 4 s value PT, but PT was still 29 ± 7% above the unpotentiated value after 45 s. Interestingly the following MVCs showed similar PT at 4 s after MVC, but PT was significantly (p<0.05) higher 30 and 45 s after MVC 2 and 8, 12, 16, 30 and 45 s after MVC 5 and 10 compared to MVC 1, indicating a slower decay of PT (Figure 3a). In addition PT at 45 s after the first MVC was significantly lower (p<0.05) than were the values 45 s after any of the following MVCs (2–10).

All potentiated values for RTD and RR were significantly higher than pre-MVC values for the duration of the experiment (Figures 3b and 3c). RTD and RR increased by 82 ± 29% and 59 ± 22% respectively after MVC 1. RTD after MVCs 5 and 10 was significantly higher (p<0.05) than after MVC 1. RR was not significantly different (p>0.05) between MVCs 1–10.

Although ½RT was not significantly different from pre-MVC values at any time during the experiment, CT was significant lower after MVCs 1–10 compared to the value measured in unpotentiated muscle, except for 30 and 45 s after MVC 1 (Figure 3b). EMD did not change at any time during this part of the experiment. Correlation between changes in PT, RTD and RR in studies 1 and 2

**Figure 4:**
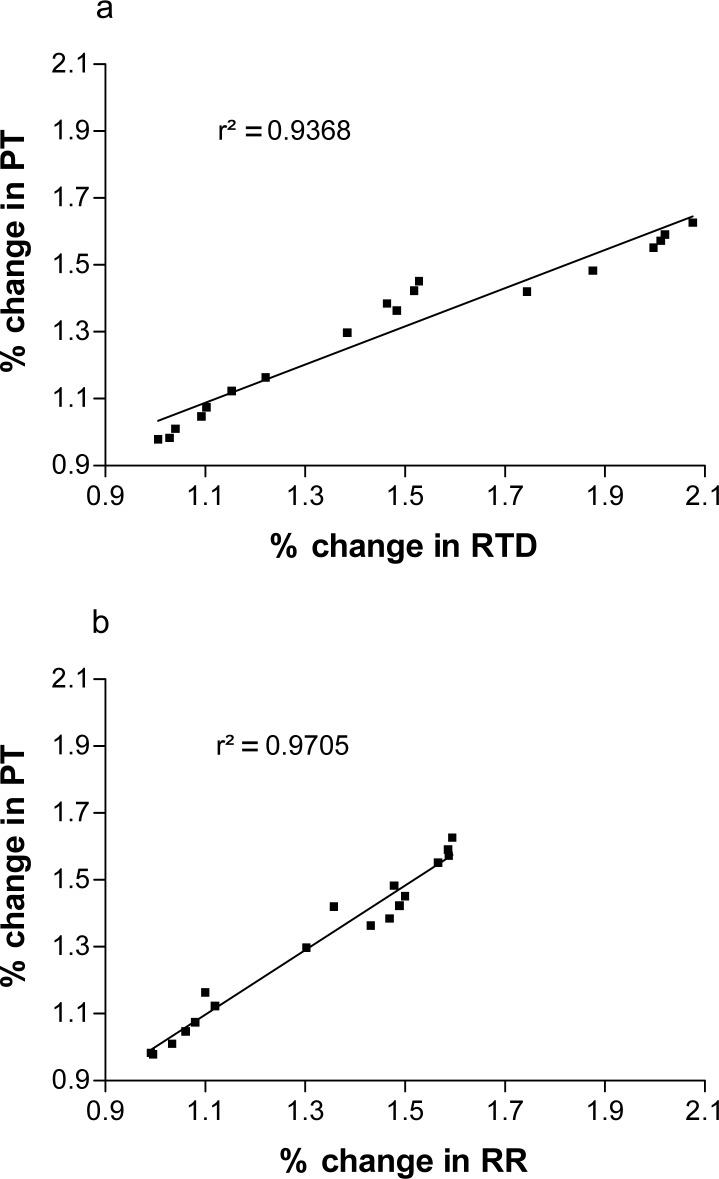
Correlation between percent change in PT and RTD (a), and PT and RR (b) for all measurements in studies1 and 2. PT, peak twitch torque. RTD, rate of torque development. RR, rate of torque relaxation.

Figure 4 shows the correlation between percentage change in PT vs. RTD and PT vs. RR for all time points for both study 1 and 2. This proves that changes in PT are significantly correlated with changes in both RTD (r^2^ = 0.94, p<0.001) and RR (r^2^ = 0.97, p<0.001).

## Discussion

Peak twitch torque is commonly used to evaluate peripheral fatigue, and we have carefully studied potentiation and decay of potentiation after 5 s MVC during repeated measurements to understand its effects on peak twitch torque. The first finding of this study was that electrical stimulation repeated 4 times every min for 10 min (Table 1) or repeated 6 times in 1 min without previous maximal voluntary contraction (Table 2) showed similar responses of peak twitch torque. This means that repeated single stimulus does not cause neither potentiation nor fatigue. Nor did repeated electrical stimulation produce any changes in measures of skeletal muscle contraction or relaxation such as RTD, RR, CT, ½RT and EMD. This finding is important because it shows that repeated single stimuli for measurements of muscle function do not alter the response to a following stimulus.

In the present study a single MVC lasting 5 s produced an instantaneous increase in peak twitch torque by 45 ± 13% (study 1) and 56 ± 10% (study 2) compared to the initial measurement (Figures 2a and 3a). These results are not different from those of Green and Jones (58%), Paasuke et al. (30–51%) and Requena et al. (48%) (Green and Jones, 1989; Paasuke et al., 2007; Requena et al., 2008), but slightly lower than the increases reported by Hamada et al. (71%), Folland et al. (67%) and Miyamoto et al. (∼100%) (Hamada et al., 2000; Folland et al., 2008; Miyamoto et al., 2012). The reason for the difference in potentiation in these studies is not clear. However, it has been reported that type 2 muscle fibres have higher potentiation than type 1 muscle fibres, and potentiation varies between individuals (Hamada et al., 2000). It is also possible that a single 5 s MVC did not produce full potentiation, since we observed higher peak twitch torque after two or more MVCs repeated every min (Figure 3a, discussed below).

Peak twitch torque in potentiated quadriceps muscles decayed exponentially and was no longer significantly different from the pre-MVC unpotentiated value after 2 min (Figure 2a) and peak twitch torque returned to baseline values only 6 min after the MVC. This is the first study to report the decay of potentiation after a 5 s isometric MVC of the quadriceps. The decay in peak twitch torque after a single 5 s MVC was slightly faster than other studies of the quadriceps (Green and Jones, 1989; Hamada et al., 2000; Requena et al., 2008) and other muscles (Vandervoort et al., 1983; Baudry and Duchateau, 2004). However the decay was slower after 5 s MVCs repeated every min. These data suggest that a single 5 s MVC is not sufficient to maximize potentiation and peak twitch torque was higher at 8–45 s after the fifth MVC. Furthermore, the effect of potentiation has a half-time of about 60 s, and it is therefore crucial that electrical stimulation is applied immediately after termination of the activity to optimize the measurement of peripheral fatigue since potentiation will start to recover and “peripheral fatigue” will start to decay immediately after termination of activity (Froyd et al., 2013). Therefore, careful characterization of development of potentiation and the decay of potentiation is necessary to design studies to investigate peripheral fatigue.

5 s MVCs repeated up to 10 times in 10 min did not cause any additional significant increase in peak twitch torque (Figure 3a). It has been reported that the first and second MVCs produced 7 and 3% lower peak twitch torque respectively than the following MVCs (Kufel et al., 2002). Indeed peak twitch torque tended to increase for the first 5 MVCs. It may be that one 5 s MVC does not maximally potentiate the muscle so that a train of more MVCs may be required for full potentiation before exercise. However, the more important finding was that the decay of potentiation was slower after the second MVC performed 55 s later. This suggests that the effect of an MVC does not completely disappear after 55 s, and an additive effect is registered. The mechanisms for potentiation have been suggested to involve phosphorylation and increased Ca^2+^ sensitivity (Vandenboom et al., 1993; Sweeney et al., 1993). In the present study, torque was elevated by 29% 45 s after the first MVC, what supported the fact that some effects of the previous MVC were maintained. The fact that the decay was slower and remained stable during the 9 following MVCs, suggests that more than one MVC should be performed prior to exercise if 5 s MVCs are used for pre-exercise potentiation in exercise studies.

Changes in peak twitch torque correlated significantly with changes in RTD and RR (Figure 4) as shown previously (Paasuke et al., 2007; Requena et al., 2008). However, we show that peak twitch torque is correlated with RTD and RR throughout the experiment. It is suggested that during fatigue, the tension decrease is caused by reduced Ca^2+^ release, Ca^2+^ sensitivity and Ca^2+^ pumping (Westerblad and Allen, 1991). This suggests that the phenomenon of potentiation as during fatiguing exercise is caused by immediate changes in measures of skeletal muscle contractility and relaxation. Further studies should be performed to explore this phenomenon.

In summary, we conclude that peak twitch torque increases immediately after a 5 s MVC. This is the first study to show that potentiation decays within 2–6 min after a 5 s isometric MVC of the quadriceps muscles, but with a tendency of faster decay than after MVCs of 7–10 seconds in other studies. We also showed that changes in peak twitch torque are related to changes in both RTD and RR. This suggests that the phenomenon of potentiation is related to immediate changes in measures of skeletal muscle contractility and relaxation.

## Figures and Tables

**Table 1:** Responses of single stimulus at specific time points at rest for study 1 (n= 5).

	4 s	*8 s*	12 s	16 s	30 s	1 min	2 min	3 min	4 min	6 min	8 min	10 min
PT Nm	43.5 ± 6.9	43.3 ± 7.6	43.5 ± 7.9	43.7 ± 7.7	44.0 ± 8.4	44.0 ± 7.8	44.3 ± 8.0	43.6 ± 7.8	44.2 ± 7.9	43.7 ± 7.5	42.8 ± 7.7	42.7 ± 7.1
EMD ms	32.0 ± 6.9	31.9 ± 6.9	32.0 ± 7.3	32.2 ± 7.0	31.9 ± 7.0	31.9 ± 7.2	31.7 ± 6.9	30.3 ± 7.0	31.8 ± 7.3	31.9 ± 7.0	31.8 ± 7.1	32.0 ± 7.1
CT ms	91.4 ± 14.7	90.0 ± 13.5	88.4 ± 13.5	88.7 ± 13.0	88.6 ± 12.9	88.3 ± 13.8	88.6 ± 12.9	89.3 ± 13.2	88.2 ± 12.6	89.1 ± 13.6	88.8 ± 12.7	88.7 ± 13.1
RTD Nm/ms	0.49 ± 0.11	0.49 ± 0.11	0.50 ± 0.12	0.51 ± 0.12	0.51 ± 0.12	0.51 ± 0.13	0.51 ± 0.12	0.50 ± 0.11	0.51 ± 0.11	0.50 ± 0.12	0.49 ± 0.11	0.49 ± 0.11
RR Nm/ms	0.55 ± 0.16	0.55 ± 0.16	0.57 ± 0.19	0.60 ± 0.21	0.61 ± 0.21	0.61 ± 0.21	0.61 ± 0.20	0.60 ± 0.19	0.60 ± 0.18	0.59 ± 0.17	0.58 ± 0.18	0.58 ± 0.16
½RT ms	80.6 ± 10.2	80.0 ± 8.9	78.3 ± 10.4	76.0 ± 12.3	74.8 ± 12.3	74.6 ± 12.9	75.8 ± 11.6	74.3 ± 10.5	75.4 ± 10.0	76.0 ± 11.1	75.5 ± 9.9	75.9 ± 10.2

PT, peak twitch torque; Nm, newton meter; EMD, electromechanical delay; ms, milliseconds, CT, contraction time; ; RTD, rate of torque development; RR, rate of relaxation; ½RT, half relaxation time. There was no significant difference between any variable.

**Table 2:** Responses of single stimulus at specific time points at rest for study 2 (n= 6)

	4 s	*8 s*	12 s	16 s	30 s	45 s
PT Nm	43.2 ± 12.2	43.6 ± 13.2	43.4 ± 13.1	42.8 ± 12.6	43.6 ± 13.1	44.1 ± 13.1
EMD ms	34.3 ± 3.0	34.0 ± 3.3	34.3 ± 3.4	34.6 ± 3.3	34.3 ± 2.8	33.7 ± 3.3
CT ms	87.5 ± 10.3	85.1 ± 10.8	90.4 ± 14.2	83.8 ± 8.4	82.3 ± 9.0	86.2 ± 10.1
RTD Nm/ms	0.51 ± 0.17	0.52 ± 0.17	0.50 ± 0.18	0.52 ± 0.18	0.54 ± 0.18	0.53 ± 0.19
RR Nm/ms	0.57 ± 0.22	0.54 ± 0.24	0.58 ± 0.23	0.53 ± 0.20	0.55 ± 0.22	0.57 ± 0.21
½RT ms	78.1 ± 11.4	84.9 ± 17.3	77.0 ± 11.9	82.0 ± 10.8	81.3 ± 12.9	78.8 ± 8.1

PT, peak twitch torque; Nm, newton meter; EMD, electromechanical delay; ms, milliseconds, CT, contraction time; ; RTD, rate of torque development; RR, rate of relaxation; ½RT, half relaxation time. There was no significant difference between any variable.
